# Clinical Effectiveness of Continuous Infusion Flucloxacillin in the Outpatient Parenteral Antimicrobial Therapy (OPAT) Setting in a UK Hospital: A Service Evaluation

**DOI:** 10.3390/antibiotics13020153

**Published:** 2024-02-03

**Authors:** Annette Margaret Clarkson, Susan Snape

**Affiliations:** 1 Pharmacy Department, Nottingham University Hospitals NHS Trust, Nottingham NG7 2UH, UK; 2 Microbiology Department, Nottingham University Hospitals NHS Trust, Nottingham NG7 2UH, UK; susan.snape@nuh.nhs.uk

**Keywords:** flucloxacillin, OPAT, continuous infusion, *Staphylococcus aureus* bacteraemia, MSSA, elastomeric device

## Abstract

The availability of stability data for the use of continuous intravenous flucloxacillin in an elastomeric device has enabled the treatment of serious Methicillin Sensitive *Staphylococcus aureus* (MSSA) in the outpatient parenteral antimicrobial therapy (OPAT) setting. This service review aimed to evaluate current standard of care to establish the clinical effectiveness and complication rates associated with its use since its introduction at our institution. A retrospective review of clinical outcomes and adverse events/complications, was undertaken for all patients who received continuous infusion flucloxacillin for complicated MSSA infection between January 2019 and July 2022 via our OPAT service. Thirty-nine patients were included. An OPAT treatment outcome of ‘Treatment aim attained uncomplicated’ was achieved in 29/39 (74%) patients. Two patients had an OPAT treatment outcome of treatment aim not attained, both of which required unexpected hospital re-admission. An adverse event/complication occurred in 8 patients. There were two relapses in the 12-month follow-up period. Our review supports the assertion that continuous infusion flucloxacillin is clinically effective and well tolerated for the treatment of complicated MSSA infection in the OPAT setting.

## 1. Introduction

Outpatient Parenteral Antimicrobial Therapy (OPAT) services facilitate treatment with intravenous (IV) antimicrobial therapy in non-inpatient settings such as clinics or patient homes. This allows patients who would otherwise be hospital inpatients to receive their necessary treatments as outpatients. Prior to the introduction of continuous intravenous infusions (CIVs), administration of flucloxacillin for serious Methicillin Sensitive *Staphylococcus aureus* (MSSA) infection was not logistically practical in the OPAT setting, due to its required dosing frequency of every 4–6 hours to ensure pharmacokinetic/pharmacodynamic (PK-PD) parameters are achieved [1]. Other once daily treatment options, namely ceftriaxone, are not favoured as a first-line treatment option for MSSA bacteraemia compared to anti-staphylococcal penicillins, given concerns of higher risk of treatment failure and conflicting results relating to efficacy in the OPAT setting [2,3]. Continuous antimicrobial infusions are advised within the OPAT good practice recommendations [4] to be used where there are robust drug stability data, such data is published for flucloxacillin [5]. 

Previous small studies in the outpatient setting have shown it to be safe and effective [6,7,8,9]. Flucloxacillin CIVs using an elastomeric device were introduced at our institution in 2019. This service review aimed to evaluate our current standard of care and to establish the clinical effectiveness and complication rates associated with the use of continuous infusion flucloxacillin in patients with proven complicated MSSA infection. Compared with other prior studies, we reviewed outcomes at a longer 12-month follow-up period.

## 2. Materials and Methods

The Nottingham OPAT service provides intravenous antibiotics to patients who have been treated at Nottingham University Hospitals NHS Trust and local community. The service assesses patients on an individual basis to ascertain the clinical need for antibiotics and the most suitable method of antibiotic administration. If intravenous antibiotics are necessary, patients are either taught to self-administer, attend the OPAT infusion centre daily, or are visited in their home and receive healthcare worker-delivered antibiotics. In 2023, there were 408 patient episodes, of which 130 were admission avoidance and 278 were early discharge. The total number of bed days saved was 11,075.

We conducted a retrospective service evaluation of all adult patients managed via the Nottingham University Hospitals NHS Trust OPAT service who received a continuous infusion of flucloxacillin between January 2019 and July 2022. Inclusion criteria were as follows: all patients prescribed a continuous 24 hour intravenous infusion of flucloxacillin and met the inclusion criteria for suitability for discharge to the OPAT service as detailed in Appendix A Table A1. All patients consented to receive treatment via OPAT. Oral antibiotics prescribed in conjunction with the continuous intravenous flucloxacillin infusion were permitted. Patients were excluded if they did not receive continuous infusion flucloxacillin as their primary intravenous antibiotic treatment at the point of discharge to the OPAT service. 

All patients received multiple daily dosing of flucloxacillin during their inpatient stay and were converted to the same total daily dose at the point of discharge. Flucloxacillin 8 g/24 h infusion (2 g every 6 h) or 12 g/24 h infusion (2 g every 4 h). The dosing and duration of treatment was dependent upon the infective indication, weight, and was decided by an infection specialist (Medical Microbiologist or Infectious Diseases Physician) specialising in OPAT. All patients had established venous access, either via a peripherally inserted central catheter (PICC) or a Midline. PICC lines inserted have a 1-year lifespan, as do 4French (4F) Midlines and were inserted by interventional radiology. 2French (2F) Midlines have a lifespan of 28 days and were inserted by an appropriately trained OPAT nurse or at ward level by an appropriately trained staff member. 

Flucloxacillin continuous infusions were prepared by Nottingham University Hospital’s Pharmacy Department sterile production unit or by Lloyds Homecare (Harlow, UK). Flucloxacillin solutions were made to a final volume of 230 mL 0.3% *w*/*v* citrate buffered sodium chloride 0.9% in a Vygon Accufusor (WooYoung Medical, supplied by Vygon, Swindon, UK) or Baxter LV10 infusers. Manufacturing complied with yellow cover document standards [5,10]. Infusions were stored in the fridge (2–8 °C) prior to use and were removed from the fridge 2 hours prior to connection. Patients were either trained to self-administer, attend the OPAT Infusion Centre daily, or an OPAT nurse attended their home to change the continuous infusion on a daily basis. All patients received a 2 g loading dose of flucloxacillin, given as a slow bolus prior to connection of the first continuous infusion. Patients had baseline blood tests (urea and electrolytes (U&Es), full blood count (FBC), liver function tests (LFTs), C-reactive protein (CRP), calcium, Vitamin D, and international normalised ratio (INR)). U&Es, FBC, LFTs, and CRP were checked weekly thereafter. All patients received line care weekly and were reviewed by a multi-disciplinary team in a virtual ward round on a weekly basis and regularly reviewed in clinic. Table 1 summarises the follow-up care and investigations performed for all patients.

Patients were identified from the OPAT patient management system (OPAT PMS) or from electronic copies of prescriptions for continuous flucloxacillin infusions. Individual patient records on the OPAT PMS, hospital record systems (NotIS and Medway), and microbiology records (Winpath) were then retrospectively reviewed. The following data were collected: patient demographics, co-morbidities, indication for antibiotic therapy, dose, duration, method of venous access, positive microbiology specimens, additional anti-infective treatments, method of administration (self-administered, nurse delivered), side effects/reasoning for early discontinuation, and laboratory results to assess any adverse effects. The data were collected and analysed in Microsoft Excel (2016).

At the beginning of treatment on OPAT, patients were prospectively assigned a treatment aim of cure, improvement, or palliation. Treatment aim definitions are in accordance with the UK OPAT good practice recommendations [4] and are detailed in Appendix B, Table A2. 

Clinical and microbiological outcomes were assessed at the end of treatment and 12 months after. Outcomes at the end of OPAT treatment were retrieved from the OPAT PMS, which prospectively records the OPAT outcome as defined by the UK OPAT good practice recommendations [4] and are detailed in Appendix B, Table A2. Follow up after 12 months was conducted by a retrospective review of hospital records to ascertain if there had been any re-admissions or positive microbiology associated with likely relapse of the initial infection. 

An adverse event of acute kidney injury (AKI) was determined through comparison with a change in laboratory values over time. Our local laboratory defines AKI based on comparison of the current result compared with a previous result and are aligned with the kidney disease improving global outcome (KDIGO) criteria. An AKI stage 1 being defined as a 1.5–2 times increase versus baseline or an increase of >25 µmol/L within 48 h. AKI stage 2 defined as a 2–3 times increase compared to baseline. 

## 3. Results

Thirty-nine patients were reviewed; the majority of patients were male (82%). The mean age was 66 years (range 39–90 years). Patient weights ranged from 48.4 to 169.7 kg, with 18 patients weighing >85 kg and 21 patients weighing <85 kg. Thirty-five patients (89.7%) had an underlying illness, and details of the number of co-morbidities exhibited by each patient are detailed in Table 2.

Of those with a co-morbidity, the number of patients with each underlying illness is identified in Table 3. Hypertension and diabetes were the most common. 

### 3.1. Intravenous Access and Method of Administration

Thirty-three patients (84.6%) had central line access via a PICC, five patients had a 4F Midline and one patient had a 2F Midline. Twenty-one patients attended the OPAT infusion centre on a daily basis for device change, three patients self-administered, and fifteen patients received therapy via a nurse visiting the patient’s home. 

### 3.2. Microbiology

The main causative organism was methicillin sensitive *Staphylococcus aureus* (MSSA), with 27 patients presenting with an MSSA bacteraemia. One patient presented with a bacteraemia secondary to *S. lugdunensis* and one with a bacteraemia secondary to a Coagulase Negative Staphylococcus (CoNS).

Of the 27 patients with an MSSA bacteraemia, 6 also had positive samples from other sites 5 deep and 1 superficial. Nine patients who were not bacteraemic grew MSSA from other sites (7 deep and 2 superficial). One patient did not have any positive microbiology and was treated for septic arthritis of the knee, where the presumed most likely pathogen was MSSA, and they responded to the treatment given.

### 3.3. Flucloxacillin Dosing and Adjunctive Oral Therapy

Twenty patients received an initial dose of 8 g/24 h and nineteen received 12 g/24 h. One patient received 8 g initially, which was increased to 12 g after 6 weeks due to lack of clinical improvement secondary to inadequate source control. This patient was receiving treatment for multi-focal MSSA infection (discitis, epidural abscess, and right psoas collection). Twenty-three patients received an oral antibiotic in conjunction with the continuous flucloxacillin infusion, which was rifampicin (17 patients), sodium fusidate (2 patients), clindamycin (2 patients), and metronidazole (2 patients). One patient received oral rifampicin initially and then changed to sodium fusidate secondary to nausea, which was also stopped due to continued nausea despite anti-emetics. The patient with multi-focal MSSA infection (described above) initially received clindamycin for 6 weeks before changing to sodium fusidate alongside the increased dose of flucloxacillin. 

### 3.4. Diagnoses and Treatment Outcomes

Patients were treated for a range of infective diagnoses as detailed in Table 4. Thirty-eight patients had a treatment aim of cure. One patient being treated for vascular graft infection had a treatment aim of palliation. Two patients failed treatment, recorded as OPAT outcome ‘Treatment aim not attained’. Both of these patients were being treated for complicated MSSA infection with discitis plus other sites.

One patient who was treated for an infected elbow replacement, relapsed 1 month following completion of 6 weeks IV and 6 weeks oral treatment, which was attributed to the re-introduction of methotrexate for arthritis.

One patient died 4 months following completion of treatment, this was due to a Gram-negative bacteraemia on a background of known hepatocellular carcinoma and the death was considered unrelated to OPAT treatment. 

### 3.5. Adverse Effects and Complications

Three patients had acute kidney injury (AKI) whilst receiving flucloxacillin. One patient was being treated for endocarditis and was subsequently changed to cefazolin and went on to successfully complete treatment. For one patient, the AKI was not significant enough to require a change to therapy and they were able to complete treatment with extra monitoring of renal function. The final patient (requiring treatment for discitis) developed AKI 24 days into treatment (they had received 11 days flucloxacillin prior to discharge on OPAT). Flucloxacillin was stopped and the patient successfully completed the remaining 3 weeks of treatment on high-dose (600 mg four times a day) oral clindamycin alone. There were no cases of hepatotoxicity or haematological side effects (neutropenia, leucopenia, thrombocytopenia) observed.

One patient developed an axillary vein thrombosis 5 days following the insertion of a 4F Midline to facilitate OPAT treatment. The 4F Midline was removed and replaced with a new 5F Midline and the patient completed the 2 weeks total IV therapy as planned. The patient was prescribed 3 months anticoagulation therapy with apixaban. 

One patient developed a maculopapular itchy rash 17 days into flucloxacillin treatment (they had received flucloxacillin as an inpatient for 14 days prior to discharge). They were switched to Ceftriaxone for the remainder of the treatment without incident.

One patient being treated for necrotising otitis externa based on an ear swab which isolated MSSA ceased treatment with flucloxacillin after 3 weeks, as a repeat ear swab isolated *Pseudomonas aeruginosa*. They were subsequently switched to piperacillin tazobactam.

One patient developed a red, swollen, and hot arm 2 days post line insertion, whereafter the PICC line was removed and a new PICC line inserted. Thrombosis was excluded, and the patient received treatment with oral doxycycline for 7 days for catheter-associated phlebitis. 

Table 4 details a full summary of dosing, clinical outcomes at the end of the OPAT treatment course, and outcomes at 12-month follow-up sorted by each infective indication. 

## 4. Discussion

1. The use of continuous infusion elastomeric devices in the OPAT setting has expanded in recent years due to the increasing availability of stability data [11]. Stability data for flucloxacillin in particular [5] enables patients to receive a narrow-spectrum first-line treatment option for serious MSSA infection, which complements the antimicrobial stewardship agenda [12]. Previous studies reviewing the use of continuous infusion flucloxacillin for complex MSSA infection in the OPAT setting have shown that sufficient flucloxacillin concentrations are achieved and have shown it to be well tolerated with high cure rates [6,7,8]. Favourable outcomes have also been shown by other OPAT centres in the UK, using alternative outcome measures [9];
2. Overall, 95% (n = 37) of patients reviewed had an OPAT outcome of ‘treatment aim attained’, though this was complicated in 8 patients. A successful outcome of ‘Treatment aim attained uncomplicated’ was observed in 29/39 (74%) patients. Thirty patients (77%) had an MSSA bacteraemia and there were a significant number of particularly difficult-to-treat infections with discitis (alone or in conjunction with infection at other sites) and endocarditis being the predominant diagnoses (44%, 17 patients). A systematic review [13] showed an overall mortality rate at 3 months of 27% and 30% at 1 year for *S. aureus* bacteraemia, with a higher mortality rate amongst those with underlying co-morbidities. In our cohort, there were no deaths within the 1-year follow-up period secondary to recurrence of MSSA infection. Further to the supportive data explained above, a low relapse rate of 5% (n = 2) within 1 year after treatment completion, in a cohort where 41% of patients had three or more underlying co-morbidities, provides further supportive evidence that the use of CIV flucloxacillin seems to be an effective treatment option for complicated MSSA infection in the OPAT setting. It is recognised that all patients in this cohort received a minimum of 2 weeks IV flucloxacillin therapy and were clinically stable prior to discharge on OPAT, and so this may be reflective of the satisfactory clinical outcomes attained. Direct comparison with other studies showing CIV flucloxacillin to be effective [6,7,8] is complex due to the differing number of diagnoses and differences in co-morbidities exhibited by the patient population;
3. There were only two patients (5%) with an OPAT outcome of ‘treatment aim not attained’, in both cases, the patients were being treated for ‘discitis plus other sites’. One patient had disseminated MSSA bacteraemia, with a left shoulder septic arthritis, multi-level discitis, posterior epidural abscess, and a right, upper lobe cavitating pneumonia. The patient was re-admitted 22 days prior to the end of his planned 6 weeks total treatment due to increasing back pain. Repeat blood cultures were negative, and MRI showed no change to epidural abscess, but some new oedema at T5. The patient completed a total of 8 weeks IV flucloxacillin and was discharged with a further 4 weeks oral treatment with doxycycline and rifampicin, completing 12 weeks treatment in total. A 12-week treatment duration is not out of keeping with recommended treatment durations required to manage discitis, given difficulties with antibiotic penetration into discs. Whilst a randomised controlled trial showed non-inferiority for a treatment duration of 6 versus 12 weeks in the treatment of vertebral osteomyelitis, non-inferiority was not shown in all subgroups and the presence of *S. aureus* infection showed reduced chances of treatment success [14]. The second patient, treated for discitis, epidural abscess, and a right psoas collection, was re-admitted 3 weeks following the completion 6 weeks IV flucloxacillin (8 g) plus oral clindamycin. Repeat imaging showed disease progression requiring source control. The patient subsequently received a further 12 weeks of IV flucloxacillin (12 g/24 h) plus oral fusidic acid, with no evidence of relapse at 12 months. Treatment failure was considered secondary to inadequate source control rather than a failure of flucloxacillin. Interestingly, this patient was bacteraemic with the same organism (MSSA) 8 weeks prior to the initial episode described above and received treatment for endocarditis (2 weeks IV flucloxacillin followed by 4 weeks IV ceftriaxone on OPAT). The choice of ceftriaxone for the initial MSSA bacteraemia may be challenged by some given the concerns about the higher risk of treatment failure compared to anti-staphylococcal penicillins [2,3];
4. In our patient cohort, one patient received a prolonged course of IV antibiotics for 6 weeks out of a total 12-week duration for prosthetic joint infection (debridement, antibiotics and implant retention (DAIR)). Whilst the prolonged intravenous duration may be seen as a treatment failure, given the outcomes published in the oral versus intravenous antibiotics for bone and joint infection (OVIVA) study [15]; the use of high-dose intravenous therapy aimed to provide the patient with the best chance of treatment success, given the multiple prior joint revisions on a background of immunosuppression;
5. The low relapse rate of 5% (n = 2) within a significant follow-up period of 12 months is encouraging. Furthermore, relapse in both cases was not deemed due to a failure of flucloxacillin treatment. One relapse was on a background of multiple joint revisions and the re-introduction of immunosuppressant therapy and the other due to a new PICC line. Two patients required surgery within the 12-month follow-up period, which were not deemed a relapse, as surgery was an expected outcome prior to commencement of antibiotic therapy in both cases;
6. Excluding the line related complications observed in two (5%) patients (axillary vein thrombosis and line related phlebitis), flucloxacillin was well tolerated by the majority of patients (87%, n = 34). Within our patient cohort, two required cessation of flucloxacillin due to suspected induced nephrotoxicity (normalisation of renal function was achieved upon cessation of therapy). This is a higher rate than that observed in other studies [6,7,8] where this adverse event was not observed. The incidence of nephrotoxicity is reported as very rare in accordance with the manufacturer’s product information [16]. It is, however, not an unexpected adverse effect [17,18] and reflects the importance of frequent monitoring of renal function in patients on prolonged treatment courses. Use of prolonged beta lactam infusions in an observational study did not show any significant increased risk of AKI versus intermittent dosing, though it is noted that this study did not include flucloxacillin [19];
7. There are some disadvantages to the use of continuous infusion elastomeric devices including leakage and residual volumes, resulting in patients failing to receive the full prescribed dose. Residual volumes have been anecdotally reported by numerous OPAT centres, with piperacillin tazobactam often being more problematic compared to flucloxacillin [20,21]. In this patient cohort, there were no reports of leakage or incomplete infusion with any of the elastomeric flucloxacillin infusions. Whilst a retrospective review of nursing documentation was not undertaken as part of this evaluation, it is standard practice for the OPAT nurses to record the time and date the infusion was connected and the time and date of disconnection. Furthermore, the infusion volume remaining is documented, and it is standard practice for the OPAT nurse to report any issues around leakage or residual volumes to the OPAT pharmacist and to document on the OPAT PMS. The OPAT PMS was reviewed as part of this evaluation, and no documentation of incomplete infusion was recorded for any of the patients receiving continuous infusion flucloxacillin in this cohort. At our OPAT centre, our experience is that residual volumes are more problematic with piperacillin tazobactam. Due to the increasing frequency of residual volumes, particularly with 18g piperacillin tazobactam 24 hour infusions, our OPAT centre introduced the administration of extra bolus doses should there be greater than a 60 mL remaining infusion volume in May 2022. In all cases reasons for incomplete infusion are investigated. For example, line obstruction, inappropriate positioning, or storage of the device, and if remaining volumes continue to be problematic, alternative antibiotic therapy is sought.

## 5. Limitations

The small sample size and in particular the lack of a control group makes conclusions about the effectiveness of continuous infusion flucloxacillin challenging, especially given the varied patient demographics, co-morbidities, and severity of infection. A case-controlled cohort study of continuous infusion flucloxacillin versus intermittent dosing (4–6 times a day) in the outpatient setting, however, would not be possible, as self-administration of flucloxacillin (4–6 times a day) is too overwhelming for patients and an OPAT nurse is unable to attend a patient’s home this frequently. This retrospective review aimed to evaluate outcomes based on current recommended clinical practice in the OPAT setting, and therefore did not compare clinical outcomes versus intermittent dosing flucloxacillin.

Flucloxacillin concentrations were not measured as a proxy of effectiveness, as have been described in other studies [6,7,8]; however, the high success rate and low relapse relate we observed are suggestive that sufficient drug concentrations were present to achieve clinical cure. Whilst the follow-up period of 12 months is longer than the 3 months in other studies [6,8], records of relapse or re-admission due to treatment failure were only taken from our hospital records, and therefore would not have included re-admission to other hospitals. However, given the complexity of the infections treated and that our hospital is a regional specialist centre, it would be expected for these patients to have been re-referred back to the specialist team who made referral to the OPAT service.

## 6. Conclusions

Despite the small sample size, this review adds further support and contributes to an expanding body of evidence to suggest that continuous intravenous flucloxacillin can be effective and well tolerated in the treatment of complicated MSSA infection in the OPAT setting. Future studies incorporating a control group comparing intermittent dosing (4–6 times daily) versus the use of continuous infusion flucloxacillin in the management of complicated MSSA infection are required, which would need to be undertaken within the inpatient setting. Further studies are particularly required to determine whether continuous infusion flucloxacillin is effective in the acute management (within the first 2 weeks) of MSSA infection in the inpatient setting compared with intermittent dosing. 

## Figures and Tables

**Table 1:** Summary of investigations and follow up for all patients whilst on the OPAT service.

Investigation/Follow Up	Frequency	Description/Further Information
Blood tests	Baseline prior to discharge on OPAT, then weekly till stop date	Baseline U&Es, FBC, LFTs, CRP, calcium, INR, and vitamin D. Weekly: U&Es, FBC, LFTs, CRP
Multi-disciplinary ward round	Weekly until stop date	Ward round attended by OPAT consultant, OPAT registrar, specialist pharmacist and OPAT nurse
Face to face clinic review	Week 1 on OPAT and at end of treatment	Face to face visits if required if problem identified
Telephone consultation	If any complications	May do telephone consultation at end of treatment, if face to face not required
OPAT nurse review	Daily/weekly	Daily if attending IVIC or OPAT nurse connecting the device. Weekly if self-administering
Line care (clean site and dressing change)	Weekly	Line removed at end of treatment
Review by referring specialty consultant	End of treatment or sooner if complications arise	If complication e.g., drainage/surgical intervention required

Abbreviations: CRP: C-reactive protein, FBC: full blood count, INR: international normalised ratio, IVIC: intravenous infusion centre, LFTs: liver function tests, U&Es: urea and electrolytes.

**Table 2:** Number of co-morbidities per patients.

Number of Co-Morbidities	No. of Patients
None	4
One	8
Two	11
Three or more	16

**Table 3:** Co-morbidities.

Co-Morbidity	No. of Patients
Hypertension	12
Diabetes (type 1 and 2)	11
Cardiac disease	8
Osteoarthritis/Rheumatoid arthritis	7
COPD ^#^/Asthma	7
Obesity (over 100 kg)	7
Atrial fibrillation	6
Mental health disorder	5
Cancer	5
Liver disease	4
Obstructive sleep apnoea	2
Aneurysm	2
Miscellaneous *	1 *

# chronic obstructive pulmonary disorder * motor neurone disease, chronic kidney disease, transient ischaemic attack, hypercholesterolaemia, previous intravenous drug user (1 patient each).

**Table 4:** Summary of clinical information, OPAT outcomes, and 12-month follow-up.

Infective Indication	No. of Patients	No. of Patients with a Bacteraemia	Daily Dose (g)	Duration on OPAT (Days)	Intended Total Duration (Weeks)	No. of Patients on Concurrent oral Therapy	Adverse Effects/Complications	OPAT Outcome	Follow-Up (Minimum 12 Months)
Discitis plus other sites *	6	6	8–12	4–31	6	4 Rifampicin 1 Clindamycin	1 AKI (stage1) 1 Rash	Treatment aim attained—uncomplicated: 2 Treatment aim attained—complicated: 2 Treatment aim not attained: 2	4 No relapse 2 required readmission ^$^
Endocarditis	7	7	8–12	6–25	4–6	3 Rifampicin	1 AKI (stage1)	Treatment aim attained uncomplicated: 6 Treatment aim attained complicated: 1	No relapse
Parotitis	1	1	8	9	2	1 Metronidazole	0	Treatment aim attained uncomplicated	No relapse
Vascular graft infection	2	2	12	24–43	6	1 Rifampicin	0	Treatment aim attained uncomplicated: 2	No relapse
Central line related	1	1	8	10	4	None	0	Treatment aim attained uncomplicated	Repeat MSSA bacteraemia and septic arthritis 6 months later.
Multi focal septic arthritis	1	0	12	14	6	1 Rifampicin	0	Treatment aim attained uncomplicated	No relapse
Necrotising otitis externa	1	0	8	21	6	None	Positive ear swab for *Pseudomonas* changed to Piperacillin/ tazobactam	Treatment aim attained complicated	No relapse
Septic arthritis	2	1	12	5–29	2–6 **	None	0	Treatment aim attained uncomplicated 2	No relapse
Discitis	4	4	8–12	13–71	6–12	1 Clindamycin 1 Rifampicin	1 AKI (stage 1) 1 Cellulitis (line site)	Treatment aim attained uncomplicated:2 Treatment aim attained complicated 2	No relapse
Surgical-related deep wound infection	1	0	12	9	2	None	Vaginal thrush Axillary vein thrombosis	Treatment aim attained complicated	No relapse
Septic arthritis and epidural abscess	2	2	8	18–22	4–6	2 Rifampicin	0	Treatment aim attained uncomplicated: 2	No relapse
Prosthetic joint infection (DAIR)	1	0	8	34	12 (6 IV + 6 oral)	1 Rifampicin	0	Treatment aim attained complicated	Relapse
Peripheral line related	1	1	8	7	2	None	0	Treatment attained uncomplicated	Died 4 months after completion unrelated
Epidural abscess	1	1	12	9	2 (IV) + 4 oral	1 Rifampicin	0	Cured Treatment attained uncomplicated	No relapse
Empyema	1	1	12	15	4	None	0	Treatment attained uncomplicated	No relapse
Osteomyelitis (surgical related)	4	0	8	2–33	6–12 #	3 Rifampicin	1 nausea	Treatment attained uncomplicated: 4	2 no relapse 1 elective below knee amputation 1 elective 2 stage revision
Osteomyelitis (non-surgical related)	3	3	8–12	10–37	6	1 Metronidazole	0	Treatment aim attained uncomplicated 3	No relapse

Abbreviations: AKI: acute kidney injury, DAIR: debridement, antibiotics, and implant retention, IV: intravenous, No.: number, g: gram, *: other sites include empyema, epidural abscess, psoas collection, pyomyositis, septic arthritis, cavitating pneumonia, septic lung emboli, **: 1 patient intended duration 2 weeks IV and 2 weeks oral, #: 1 patient intended duration 6 weeks IV and 6 weeks oral (DAIR), $: re-admitted 22 days prior to planned initial 6-week stop date, 1 patient re-admitted 3 weeks post finishing 6-week course.

## Data Availability

Data are contained within the article.

## Appendix A

**Table A1:** Inclusion and exclusion criteria for admission to the Nottingham University Hospitals NHS Trust OPAT service.

Inclusion Criteria
Antibiotic for OPAT is clinically effective for infection with appropriate antibiotic medical plan in medical notes Patient is medically, surgically, and socially fit for discharge Age 16 years or above Must have daily contact with relative/friend or carer Have telephone access Consent to treatment at home or daily hospital attendance Consent to OPAT nurse assessing suitability of home condition If the patient is to self-administer antibiotics they must: Be psychologically stable Have no significant visual impairment Have no significant problems affecting manual dexterity Consent to training to administer own intravenous antibiotics Suitable venous access device in place which is functional and reliable
**Exclusion Criteria**
Current intravenous drug user or patients who self-harm Patients with unstable co-morbidities or insurmountable barriers to effective treatment Patients with deteriorating renal function, liver function Patients who have known allergies to named treatments Patients who are medically unstable and require inpatient therapy

## Appendix B

**Table A2:** UK OPAT good practice recommendations [4] proposed treatment aims and OPAT service outcomes.

Treatment Aim/Outcome	Description
Cure	To complete an agreed OPAT duration of therapy on either intravenous and/or complicated oral antimicrobials with no requirement for long-term antimicrobial therapy
Improvement	To complete an agreed OPAT duration of therapy on either intravenous and/or complicated oral antimicrobials (a) as part of an agreed surgical infection management plan with further surgery planned or (b) where there is a requirement for subsequent long-term or an extended course of oral suppressive antimicrobial therapy, or (c) where potentially infective prosthetic material is still in situ.
Palliation	To undertake a course of OPAT on either intravenous and/or complicated oral antimicrobials where there are agreed ceilings of care due to co-morbidities, with death being the likely outcome
OPAT outcome	
Treatment aim attained uncomplicated	Completed OPAT therapy as per treatment aim with no unplanned changes in antimicrobial agent, no adverse events, no planned or unplanned readmission related to the current OPAT episode, no readmission of ≥24 h for unrelated event
Treatment aim attained-complicated	Completed OPAT therapy as per treatment aim but with one or more of the following: unplanned changes in antimicrobial agent, any adverse event including readmission for <24 h related to the current OPAT episode
Treatment aim not attained	Failure to complete planned OPAT therapy for any reason other than readmission due to unrelated event, worsening of infection requiring re-admission or readmission for ≥24 h for any cause related to OPAT, including adverse events

Abbreviations: h. hours, OPAT.: outpatient parenteral antibiotic therapy.
